# Serum metabolites predict response to angiotensin II receptor blockers in patients with diabetes mellitus

**DOI:** 10.1186/s12967-016-0960-3

**Published:** 2016-07-05

**Authors:** Michelle J. Pena, Andreas Heinzel, Peter Rossing, Hans-Henrik Parving, Guido Dallmann, Kasper Rossing, Steen Andersen, Bernd Mayer, Hiddo J. L. Heerspink

**Affiliations:** Department of Clinical Pharmacy and Pharmacology, University of Groningen, University Medical Center Groningen, P. O. Box 30.001, 9700RB Groningen, The Netherlands; emergentec biodevelopment GmbH, Vienna, Austria; Steno Diabetes Center, Gentofte, Denmark; Faculty of Health Science, University of Aarhus, Aarhus, Denmark; University of Copenhagen, Copenhagen, Denmark; Department of Medical Endocrinology, Rigshospitalet University Hospital of Copenhagen, Copenhagen, Denmark; Biocrates Life Sciences AG, Innsbruck, Austria

**Keywords:** Metabolomics, Albuminuria, ARB response

## Abstract

**Background:**

Individual patients show a large variability in albuminuria response to angiotensin receptor blockers (ARB). Identifying novel biomarkers that predict ARB response may help tailor therapy. We aimed to discover and validate a serum metabolite classifier that predicts albuminuria response to ARBs in patients with diabetes mellitus and micro- or macroalbuminuria.

**Methods:**

Liquid chromatography-tandem mass spectrometry metabolomics was performed on serum samples. Data from patients with type 2 diabetes and microalbuminuria (n = 49) treated with irbesartan 300 mg/day were used for discovery. LASSO and ridge regression were performed to develop the classifier. Improvement in albuminuria response prediction was assessed by calculating differences in R^2^ between a reference model of clinical parameters and a model with clinical parameters and the classifier. The classifier was externally validated in patients with type 1 diabetes and macroalbuminuria (n = 50) treated with losartan 100 mg/day. Molecular process analysis was performed to link metabolites to molecular mechanisms contributing to ARB response.

**Results:**

In discovery, median change in urinary albumin excretion (UAE) was −42 % [Q1–Q3: −69 to −8]. The classifier, consisting of 21 metabolites, was significantly associated with UAE response to irbesartan (p < 0.001) and improved prediction of UAE response on top of the clinical reference model (R^2^ increase from 0.10 to 0.70; p < 0.001). In external validation, median change in UAE was −43 % [Q1–Q35: −63 to −23]. The classifier improved prediction of UAE response to losartan (R^2^ increase from 0.20 to 0.59; p < 0.001). Specifically ADMA impacting eNOS activity appears to be a relevant factor in ARB response.

**Conclusions:**

A serum metabolite classifier was discovered and externally validated to significantly improve prediction of albuminuria response to ARBs in diabetes mellitus.

**Electronic supplementary material:**

The online version of this article (doi:10.1186/s12967-016-0960-3) contains supplementary material, which is available to authorized users.

## Background

Intervention in the renin-angiotensin-aldosterone system (RAAS) has convincingly shown to delay progression of renal disease in patients with diabetes mellitus with elevated urinary albumin excretion (UAE) in several large trials. However, individual patients show a large variability in long-term renoprotective response, which is linked to a large variability in the short-term response in albuminuria and blood pressure [1]. Consequently, a considerable proportion of patients still have significant residual albuminuria, which may contribute to progressive renal function loss [2]. The reasons behind these individual differences in response to therapy are not completely understood, but are in part related to renal tissue-specific RAAS activity, dietary salt intake, or genetic background [3–7], among others. Identifying novel biomarkers that predict the albuminuria lowering response to RAAS intervention may improve the current “trial-and-error” approach to choosing the optimal therapy for treatment of patients with diabetes mellitus. This would mark a step further to implementation of personalized medicine.

Biomarker discovery has advanced significantly over the past years with the use of high-throughput omics screening platforms. Omics profiling has emerged as a research area to expand beyond biomarker discovery to also unravel molecular pathways involved in disease pathophysiology. Integrating these data with clinical data may help give further insights in the underlying molecular mechanisms of drug response variability. Prospective metabolomics studies predicting disease progression in diabetes mellitus are becoming more common [8–10], but to our knowledge, there are to date no metabolomics studies for the prediction of drug response in diabetes mellitus.

Therefore, the aims of this study were to first discover and validate a serum metabolite classifier that predicts response in albuminuria to angiotensin II receptor blocker (ARB) therapy in patients with diabetes mellitus and micro- or macroalbuminuria, and secondly, to integrate the identified metabolites in a molecular process model capturing disease pathophysiology at the interface of drug mechanism of action to decipher the underlying molecular processes driving albuminuria response to ARB.

## Methods

### Patients and study design

Serum samples from patients enrolled in two distinct clinical studies conducted at the Steno Diabetes Center (Gentofte, Denmark), assessing the albuminuria lowering effect of ARBs were used for the present study. Both studies were performed in accordance with the Declaration of Helsinki and approved by the local ethical committee. All patients gave their informed consent.

For the discovery cohort, we used data from a crossover clinical study in type 2 diabetes assessing the effect of the ARB irbesartan. This cohort has been previously described [11]. In short, 52 patients with type 2 diabetes, hypertension, microalbuminuria, and treated with antihypertensive medication were recruited for a double-masked randomized crossover trial. At inclusion, previous antihypertensive treatment was discontinued and replaced with bendroflumethiazide, 5 mg once daily, for the entire study. Following 2 months wash-out (baseline), patients were treated randomly with irbesartan 300, 600, and 900 mg once daily. All treatment periods were of 10 weeks’ duration and consisted of an initial two-week dose titration period with irbesartan 300 mg once daily followed by 8 weeks treatment with irbesartan 300, 600, and 900 mg once daily in random order. For the present study, patient data and metabolomics measurements were available for 49 patients with type 2 diabetes and persistent microalbuminuria. For this discovery cohort, we defined the outcome of interest as percent change in UAE after 12 weeks of treatment of ibestartan 300 mg/day compared to baseline UAE.

For the external validation cohort, we used data from a clinical study in type 1 diabetes assessing the effect of the ARB losartan. This cohort has been previous described [12], and included patients with type 1 diabetes, hypertension, and diabetic nephropathy. After a four-week washout, the patients received 100 mg losartan once daily and were followed prospectively with a mean follow-up period of 36 months. For the present study, patient data and metabolomics measurements were available for 50 patients with type 1 diabetes and macroalbuminuria. For the validation cohort, we defined the outcome of interest as percent change in UAE from baseline to UAE after 16 weeks of treatment of losartan 100 mg/day. In this cohort, glomerular filtration rate (GFR) was measured by plasma clearance of ^51^Cr-EDTA every 6 months.

We expected the treatment effect of irbesartan and losartan on albuminuria to be fully present after 12 or 16 weeks of treatment, respectively. We refer to this as the response period.

### Metabolomics measurements

Serum metabolomics were performed blinded and measured by BIOCRATES Life Sciences (Innsbruck, Austria). Flow injection analysis and liquid chromatography-tandem mass spectrometry based targeted metabolomics measurements were performed on serum samples [13]. Samples were randomized on the plate prior to analysis to avoid potential confounding interaction between concentration and order of injection and to ensure a homogenous between-plate design in regard to study groups. The full set of 185 metabolites from the following chemical classes were quantified: acylcarnitines, amino acids, biogenic amines, energy/sugar metabolism (Hexoses), lysophosphatidylcholines, phosphatidylcholines, and sphingomyelins. The quantification of amino acids, acylcarnitines, sphingomyelins, phosphatidylcholines, hexose (glucose), and biogenic amines was performed using a AbsoluteIDQ™ *p180 kit* [14]. The assay was based on PITC (phenylisothiocyanate)-derivatization in the presence of internal standards followed by FIA-MS/MS (acylcarnitines, lipids, and hexose) and LC/MS (amino acids, biogenic amines) using an API4000 QTrap^®^ mass spectrometer (Applied Biosystems/MDS Analytical Technologies, Darmstadt, Germany) with electrospray ionization. Multiple reaction monitoring (MRM) detection was used for quantification applying the spectra parsing algorithm integrated into the MetIQ software (Biocrates Life Sciences AG, Innsbruck, Austria). Metabolites containing more than 70 % missing values across all samples were removed from analysis. Resting missing value singletons were omitted in statistical analysis. Missing values are imputed by nearest neighbor method with k = 6 by using the R package pcaMethods [15]. Measured values are log2-transformed to obtain normally distributed metabolite variables and to stabilize variance.

### Statistical analyses

Analyses were performed using SAS version 9.3. Baseline characteristics with normal distribution were reported as mean and standard deviation (SD), characteristics with skewed distribution were reported as median and 25th and 75th percentile [Q1–Q3], and categorical variables were reported as number and percentage. The natural log of UAE was used in all regression analysis.

Statistical modeling consisted of several steps using a previously described methodology for development of a classifier [16]. First, a least absolute shrinkage and selection operator (LASSO) regression model was fitted in the discovery cohort to the full metabolite set to select a subset of metabolites that best predicted UAE response to ARB therapy [17]. The LASSO is advantageous for small samples sizes because it places restrictions on the absolute sizes of the regression coefficients with a tuning parameter λ and controls for multicollinearity, thereby selecting the optimal subset of variables that best predicts the outcome. The tuning parameter was optimized by five-fold cross-validation, and bootstrap (N = 1000) was used to evaluate selection probabilities of each metabolite. Next, the metabolites selected by the LASSO were refitted in a new model using ridge regression to generate the classifier. Cross-validation was performed to select a new tuning parameter for the ridge regression model that minimized the mean square error (MSE). Finally, the classifier was validated in an external cohort by applying the betas for each metabolite and the tuning parameter as estimated from the discovery cohort.

In both the discovery cohort and the validation cohort, the added value of the classifier was evaluated by deriving the explained variation of the model (R^2^) from the MSEs in order to determine whether the biomarkers significantly improved prediction on top of a model of baseline clinical parameters (age, sex, glycated hemoglobin (HbA1c), systolic blood pressure (SBP), GFR, UAE). The area under the receiver operating characteristics (ROC) curve and integrated discrimination improvement (IDI) index were calculated to assess the discriminatory ability of the serum metabolites for a dichotomous outcome of >30 % decrease in UAE during the response period. This threshold was used based on prior work [2, 18, 19].

For the validation cohort, we also determined whether the serum metabolite classifier was able to predict change in GFR after the initial response period. Patient-specific GFR change was calculated by fitting a straight line through the GFR values after the initial response period, i.e. from week 16 to the end of follow-up using a linear regression model, as was done in the original study [12]. A dichotomous outcome for GFR change ≤ or >−3.0 mL/min/1.73 m^2^/year was created to assess the discriminatory ability of the serum metabolites for accelerated renal function decline. The threshold of −3 mL/min/1.73 m^2^ was chosen based on prior studies [20–22] and was approximately the median GFR change in this cohort (−3.4 [Q1–Q3: −5.7 to 1.4]).

### Molecular model of ARB drug mechanism of action

Identification of protein coding genes showing association with ARB mechanism of action was performed by querying NCBI PubMed and gene2pubmed. For both drugs, a PubMed search using the queries ≫ "irbesartan"[TIAB] OR irbesartan[nm] ≪ or ≫ "losartan"[TIAB] OR losartan[nm] ≪ was performed for identifying publications discussing irbesartan and losartan, respectively. Genes linked to identified publications were extracted from gene2pubmed. For irbesartan, 1471 publications associated to a total of 44 genes and for losartan 8166 publications linked to 101 genes in total were identified. The total set of 125 protein coding genes was used for deriving a mechanism of action molecular model as described in Heinzel et al. [23]. In short, molecular features were mapped on a human protein interaction network, and the induced subgraph was split into molecular process segments according to network topology. The resulting ARB mechanism of action molecular model holds 48 protein coding genes embedded in seven molecular process segments.

Interference of this ARB mechanism of action molecular model was performed with a previously identified diabetic kidney disease (DKD) molecular model holding 688 protein coding genes in 34 molecular process segments [24]. Interference was defined by an overlap of interacting protein coding genes being present in both the ARB mechanism of action molecular model and the DKD molecular model.

### Assignment of metabolites

Metabolites selected for the classifier were assessed for being part of the DKD molecular model. Metabolite-to-enzyme assignments were identified utilizing the Human Metabolome Database (HMDB) and the Kyoto Encyclopedia of Genes and Genomes (KEGG) database. Of the 185 metabolites addressed in targeted metabolomics, 114 metabolites could be assigned to at least one enzyme. The respective number for the shortlist of 21 metabolites included in the classifier on drug response is 14. For these 14 metabolites, 9 could be assigned to the molecular model representation of DKD involving 11 assigned enzymes.

## Results

Baseline characteristics are presented in Table 1. In the discovery cohort, patients were approximately 59 (standard deviation 10) years of age, mostly male (80 %), had a known duration of type 2 diabetes of 13(8) years, and median 24-h UAE was 84 [Q1–Q3: 65 to 200] mg/24 h. Median change in UAE was −42 % [Q1–Q3: −69 to −8] after 12 weeks of treatment of ibestartan 300 mg/day (Table 1).

**Table 1:** Patient characteristics

	Type 2 diabetes discovery cohort (n = 49)	Type 1 diabetes validation cohort (n = 50)
Baseline		
Age (years)	59.0 (10.0)	44.6 (8.9)
Male sex (number (%))	39 (80)	30 (60)
SBP (mmHg)	140.0 (15.4)	150.6 (17.7)
DBP (mmHg)	81.6 (8.8)	84.7 (10.9)
HbA1c (%)	8.3 (1.4)	8.9 (1.2)
HbA1c (mmol/mol)	67.2 (15.3)	73.8 (13.1)
Cholesterol (mmol/l)	5.3 (1.0)	5.2 (1.0)
HDL (mmol/l)	1.2 (0.3)	1.6 (0.5)
GFR (ml/min/1.73 m^2^)	102.3 (19.2)	86.5 (23.4)
24-h UAE (mg/24 h)	84 [65, 200]	1211 [598, 2023]
Follow-up		
Change in SBP (mmHg)	−6.4 (16.2)	−8.7 (14.3)
Change in DBP (mmHg)	−6.0 (8.8)	−5.6 (8.6)
Percent change in UAE (%)	−42 % [−69, −8]	−43 % [−62, −23]
>30 % decrease in UAE from baseline [number (%)]	31 (63)	34 (68)
GFR change after response period (mL/min/1.73 m^2^/year)	Not available	−3.8 (3.6)

Data are reported as mean ± standard deviation (SD) or number (percent) or median [25th, 75th percentile]

In the validation cohort, patients were approximately 47(9) years of age, mostly male (60 %), had a known duration of type 1 diabetes for 33(9) years, and median 24-h UAE was 1211 [Q1–Q3: 598 to 2023] mg/24 h. Median change in UAE was −43 % [Q1–Q3: −23 to −62] over 16 weeks of treatment with losartan 100 mg/day (Table 1). During approximately 3 years of follow-up, GFR change after the response period was −3.8 (3.6) mL/min/1.73 m^2^/year.

There were no significant associations between baseline characteristics and change in UAE in either the discovery or validation cohorts (Additional file 1: Table S1).

### Serum metabolite classifier

Out of the total set of 185 metabolites, 21 metabolites were selected with LASSO as best predictors of UAE response to ARB therapy in the discovery cohort. These 21 metabolites were used for the classifier. The 21 metabolites are presented in Table 2.

**Table 2:** LASSO-selection of best predictors

Metabolite	Mean estimate	Standard deviation	95 % CI	Selection percentage^a^
Asymmetric dimethylarginine (ADMA)	4.4	6.5	0, 22	52.4
Asparagine (Asp)	−3.0	7.4	−26, 0	26.2
Carnitine (C0)	−2.4	6.8	−24, 0	20.0
Acylcarnitine (C12-DC)	11.3	24.6	0, 88	31.4
Linoleoylcarnitine (C18:2)	−3.0	7.5	−27, 0	22.8
Acylcarnitine (C5:1-DC)	2.9	7.4	0, 25	23.4
Glutarylcarnitine (C5-DC/C6-OH)	3.3	7.5	0, 28	27.2
Acylcarnitine (C6:1)	3.7	9.9	0, 34	23.5
Acylcarnitine (C7-DC)	2.39	6.99	0, 25	20.6
Octanoylcarnitine (C8)	−3.0	8.8	−31, 0	20.6
Citrulline (Cit)	−2.0	4.9	−17, 0	27.5
Glutamine (Gln)	3.7	7.8	0, 28	31.8
Histidine (His)	7.5	14.0	0, 48	36.2
Lysophosphatidylcholines (lysoPC a C16:0)	−10.4	18.4	−62, 0	36.9
Lysophosphatidylcholines (lysoPC a C16:1)	−2.9	6.4	−22, 0	26.2
Phosphatidylcholines (PC aa C36:0)	4.5	7.0	0, 23	44.1
Phosphatidylcholines (PC aa C42:2)	9.7	13.8	0, 46	51.6
Symmetric dimethylarginine (SDMA)	0.3	1.0	0, 3	22.5
Spermine	−3.9	7.0	−24, 0	36.2
Tryptophan (Trp)	6.2	12.2	0, 41	35.1
Valine (Val)	3.5	8. 7	0, 31	24.4

Results from LASSO regression of 21 metabolites selected for the serum metabolites classifier, five fold cross-validation, and bootstrap resampling (N = 1000) in the discovery cohort of patients with type 2 diabetes with microalbuminuria (n = 49)

^a^The relative frequency of the marker being included in the model across 1000 bootstrap resamples

**Fig. 1:**
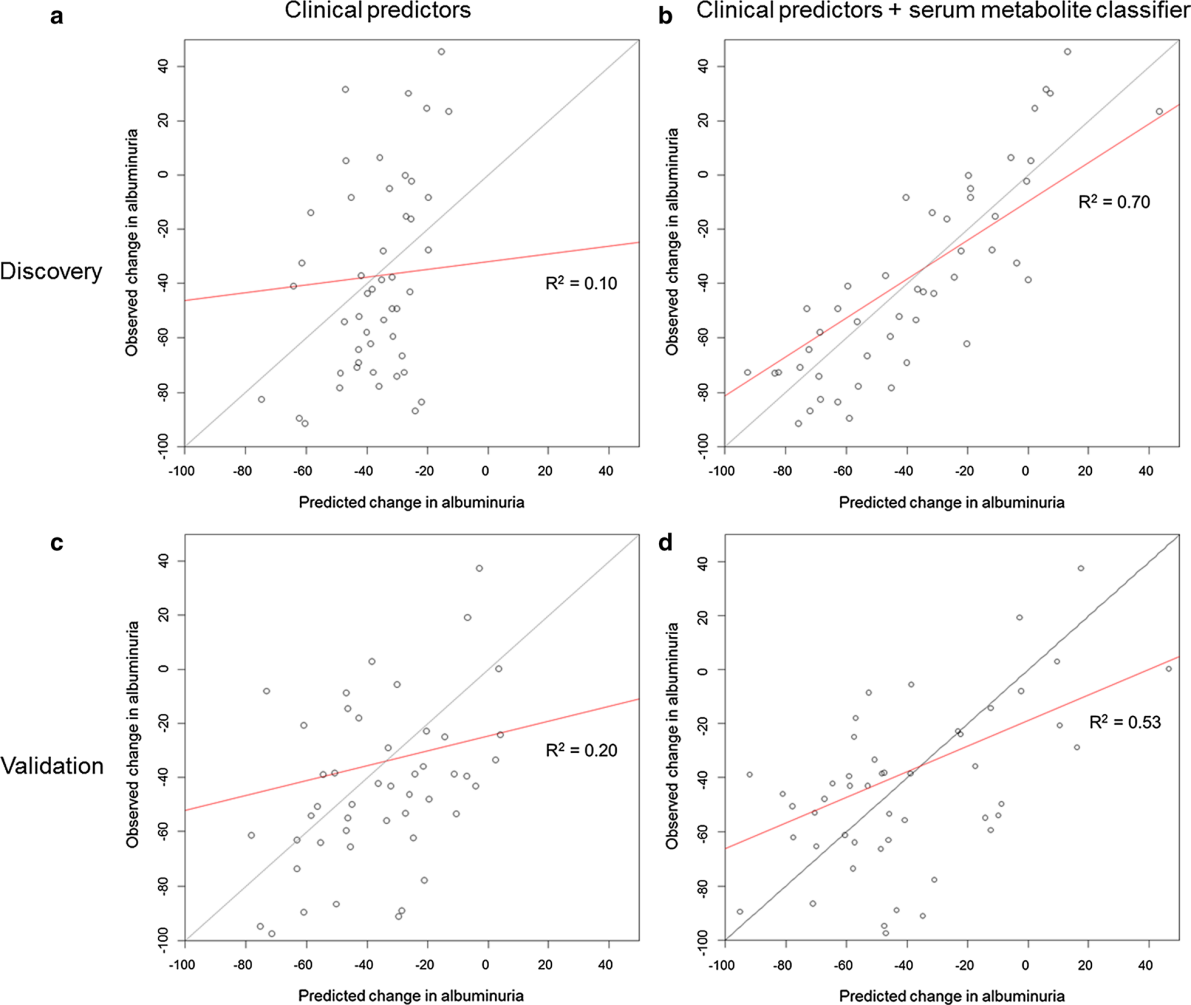
Prediction of change in UAE from baseline. **a** Discovery cohort, clinical parameters model; **b** Discovery cohort, clinical parameters + serum metabolite classifier model; **c** Validation cohort, clinical parameters model; **d** Validation cohort, clinical parameters + serum metabolite classifier model. The *lines* of identity are shown in *grey*, and the regression lines are shown in *red*. In the case of perfect prediction, the regression line would be equal to the line of identity

In the discovery cohort, the serum metabolites classifier was significantly associated with change in UAE in response to irbesartan 300 mg/day (p value <0.001) and significantly improved prediction on top of clinical parameters (R^2^ increase from 0.10 to 0.70; p value <0.001) (Fig. 1a, b; Table 3). For the dichotomous outcome of >30 % decrease in UAE during the response period, the control model area under the ROC curve was 0.72, and the addition of the serum metabolite classifier significantly increased the area under the ROC curve to 0.95 (p value = 0.001) (Table 3). The IDI of the classifier was 0.33 (p value <0.001) (Table 3). The classifier improved prediction in SBP response (R^2^ increase from 0.63 to 0.68; p value = 0.019).

**Table 3:** Risk prediction of change in UAE in response to ARB therapy

	R^2^	p value*	Discrimination of >30 % decrease in UAE							
			ROC	95 % CI		p value*	IDI	95 % CI		p value*
Discovery cohort										
Clinical parameters^a^	0.10	Ref.	0.72	0.57	0.87	Ref.		Ref.		
+Serum metabolites classifier	0.70	<0.001	0.95	0.89	1.00	0.001	0.50	0.36	0.63	<0.001
+Subset of 7 metabolites^b^	0.50	<0.001	0.90	0.81	0.99	0.012	0.33	0.19	0.47	<0.001
Validation Cohort										
Clinical parameters^a^	0.20	Ref.	0.74	0.59	0.89	Ref.		Ref.		
+Serum metabolites classifier	0.53	<0.001	0.89	0.79	0.99	0.063	0.30	0.15	0.46	<0.001
+Subset of 7 metabolites^b^	0.35	0.002	0.78	0.64	0.92	0.460	0.19	0.09	0.47	0.055

* Comparing clinical parameters + metabolites to only clinical parameters

^a^Baseline Age, Sex, SBP, HbA1c, GFR, UAE

^b^Metabolites assigned to both drug interference:direct disease phenotype and disease progression processes (seven metabolites: ADMA, citrulline, lysoPC a C16:0, lysoPC A C16:1, PC aa C36:0, PC aa C42:2, tryptophan)

In the validation cohort, the serum metabolite classifier was significantly associated with change in UAE in response to lorsartan 100 mg/day (p value <0.001) and significantly improved prediction of change in UAE on top of a panel of clinical parameters (R^2^ increase from 0.20 to 0.53; p value <0.001) (Fig. 1c, d; Table 3). For the dichotomous outcome of >30 % decrease in UAE during the response period, the area under the ROC curve for the control model was 0.74, and the addition of the serum metabolite classifier increased the area under the ROC curve to 0.89. This increase was not significant (Table 3). In The IDI of the classifier was 0.19 (p value = 0.06) (Table 3). The classifier did not improve prediction in SBP response in external validation (R^2^ control = 0.24, R^2^ classifier 0.25; p value = 0.54).

In the validation cohort, for prediction of GFR change after the response period, the combination of the 21 serum metabolites significantly improved prediction on top of clinical parameters (R^2^ increase from 0.15 to 0.60; p value <0.001). For the dichotomous outcome for GFR change ≤ or >−3.0 mL/min/1.73 m^2^/year, a significant increase was observed in the area under the ROC curve with the addition of the serum metabolites on top of clinical parameters (ROC increase from 0.71 (95 % CI 0.57 to 0.86) to 0.88 (95 % CI 0.79 to 0.98); p value 0.010).

### Metabolite assignment

**Fig. 2:**
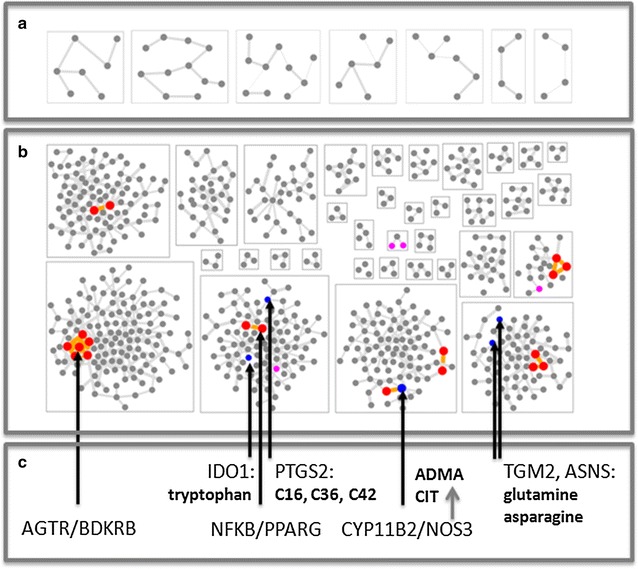
**a** Combined irbesartan/losartan drug mechanism of action molecular model holding 48 protein coding genes (*nodes*) organized in seven molecular process segments (*boxes*). Protein interactions are indicated as edges, interactions of proteins across process segments are omitted. **b** Interference of ARB mechanism of action model on the DKD molecular model. Matching network segments are shown as *red* nodes. Nodes colored in *blue* identify enzymes associated with metabolites included in the classifier, nodes in *pink* indicate metabolite transport. **c** Gene symbols for selected nodes matching in drug and DKD molecular models, and associated metabolites in case of enzymes according to Table 4

To study the molecular mechanisms linked to albuminuria response and ARB effect, molecular process analysis was conducted by assigning the metabolites included in the classifier to enzymes and further to molecular processes identified in a DKD molecular model. The combined irbesartan/losartan drug mechanism of action molecular model holding 48 protein coding genes is presented in Fig. 2a. The model interference of the ARB drug mechanism of action molecular model with the DKD molecular model is shown in Fig. 2b. In total, 20 interacting protein coding genes being reported as associated with ARB effect were also identified in the DKD molecular model. Key overlap of ARB effect with DKD pathophysiology are shown in Fig. 2c, at first including the drug target angiotensin II receptor, type 2 (AGTR2) together with the bradykinin system and the NFκB/PPARγ axis.

Nine out of the 21 metabolites included in the classifier could be assigned to eleven enzymes also involved in the DKD molecular model, including enzymatic turnover as well as metabolite transport. Metabolite-enzyme links are provided in Table 4.

**Table 4:** Metabolite-enzyme links as identified in the DKD molecular model and description of assigned reactions and transport function

Metabolite-enzyme link		Description
Metabolite	Enzyme	
ADMA	NOS2, NOS3	Produces nitric oxide (NOS) which is a messenger molecule with diverse functions throughout the body. In macrophages, NO mediates tumoricidal and bactericidal actions. Also has nitrosylase activity and mediates cysteine S-nitrosylation of cytoplasmic target proteins such COX2 ADMA is an endogeous inhibitor of eNOS (NOS3) function
Aspargine Glutamine	ASNS	Asparagine Synthase: Adenosine triphosphate + l-Aspartic acid + l-Glutamine + Water → Adenosine monophosphate + Pyrophosphate + l-Asparagine + l-Glutamic acid
Asparagine	SLC1A1	Solute carries family member 1, l-Asp cotransporter
Glutamine	TGM2	Catalyzes the cross-linking of proteins and the conjugation of polyamines to proteins
Citrulline	NOS1, NOS2, NOS3	l-Arginine + NADPH + Oxygen → Citrulline + Nitric oxide + NADP + Water NADPH + N-(o)-Hydroxyarginine + Oxygen + Hydrogen Ion → NADP + Nitric oxide + Citrulline + Water l-Arginine + Oxygen + NADPH + Hydrogen Ion → Nitric oxide + Citrulline + NADP + Water
lysoPC a C16:0 lysoPC a C16:1 PC aa C36:0 PC aa C42:2	PLA2G1B	PA2 catalyzes the calcium-dependent hydrolysis of the 2-acyl groups in 3-sn-phosphoglycerides
PC aa C36:0	ATP8A1 ATP10A PLSCR1	Transport of aminophospholipids
Tryptophan	IDO1	Catalyzes the cleavage of the pyrrol ring of tryptophan and incorporates both atoms of a molecule of oxygen

Interference of ARB drug mechanism of action molecular model and the DKD molecular model identifies the enzyme nitric oxide synthase 3 (NOS3). The metabolites included in the classifier were assigned to NOS3 are asymmetric dimethylarginines (ADMA) and citrulline, with ADMA being the most frequently selected metabolite in the LASSO (Table 2). Furthermore, overlap of enzymes at the molecular process level between the molecular models was observed involving amino acids (glutamine, asparagines and tryptophan), lysophosphatidylcholines (lysoPC a C16:0 and lysoPC a C16:1), and phosphatidylcholines (PC aa C36:0 and PC aa C42:2). This subset of seven metabolites assigned to direct drug-to-DKD interference (ADMA, citrulline) or to molecular processes with interference on the molecular model level (lysoPC a C16:0, lysoPC a C16:1, PC aa C36:0, PC aa C42:2, tryptophan) significantly improved the explained variation in albuminuria response (R^2^) in the discovery study (R^2^ = 0.50; p < 0.001 versus clinical model) and validation study (R^2^ = 0.39; p = 0.001 versus clinical model).

## Discussion

This study discovered and externally validated a serum metabolite classifier that significantly improves prediction of albuminuria response to ARBs in patients with diabetes mellitus with micro- or macroalbuminuria on top of traditional clinical risk factors. Metabolites included in the classifier could be assigned to general molecular mechanisms of oxidative stress, inflammation, and fibrosis pathways. Specifically, NOS3 activity appears to be a relevant factor in predicting the albuminuria lowering response to ARBs. Our findings suggest the use of serum metabolites as a tool to tailor albuminuria lowering ARB treatment and illustrate the use of metabolomics to unravel underlying molecular mechanisms of ARB response.

Metabolomics, the measurement of exogenous or endogenous small molecules in a sample, is an emerging research area to identify novel biomarkers. The metabolome integrates the biological information of the genome, transcriptome, proteome, and overall enzymatic reactions of an individual, therefore enabling the detection of short and long-term physiological or pathological changes occurring in diseases [25]. Metabolomics can be used to unravel molecular pathways of biological processes in order to better understand disease progression [9, 26, 27], but to the best of our knowledge, this is the first study integrating metabolomics to study response to drug therapy. These types of studies are necessary to characterize the molecular mechanisms of drug effects and drug response variability.

Pharmacological blockade of angiotensin II activity by ARBs is currently the most widely used therapeutic option, next to angiotensin converting enzyme inhibitors (ACEi), for treatment of hypertension and albuminuria in diabetes mellitus. Yet, approximately 25 % of patients with diabetes mellitus do not respond in terms of albuminuria lowering to ARBs [1]. The serum metabolite classifier was able to predict the short-term albuminuria response to the ARBs. This observation is important from a therapeutic point of view as a poor anti-albuminuric response predicts poor long-term renal prognosis [28]. Importance of early albuminuria reduction is not only evident for renoprotection but also for cardiovascular protection [2]. The metabolite panel thus provides an indication which patients will be protected for long-term renal and cardiovascular outcomes. In addition to predicting UAE response, the classifier predicted SBP response in the discovery cohort but not in the validation cohort. The failure to predict the SBP response may be explained by prior observations that not all patients experience a parallel decrease in both blood pressure and albuminuria [18, 19]. In other words, the degree of blood pressure lowering with ARB may be independent of the degree of albuminuria lowering. This has been shown in multiple studies of ARB therapy in diabetic nephropathy, such as RENAAL, IDNT, and IRMA-2 trials [1, 18, 19, 29], as well as with the albuminuria-lowering endothelin A receptor antagonist atrasentan [30]. Identification and validation of novel biomarkers that can predict response to therapy may improve on the current “trial-and-error” approach in prescribing medication, may ultimately help reduce the large individual variability in response to therapy, and could be a step forward to implement personalized medicine.

We performed a molecular process analysis aiming to further unravel the molecular mechanisms linked to albuminuria response to ARBs in diabetes mellitus. The network approach assumes that individual drug response variability can be in part attributed to individual variability in underlying molecular mechanisms involved in the progression of disease in the light of personalized molecular pathophysiology. Indeed, coupled pathophysiological processes such as oxidative stress, inflammation, and fibrosis appear to drive disease progression, but the individual contribution of each process varies per individual. Drugs on the other hand, address specific targets and thereby interfere in specific disease associated processes. At this level, metabolites, or biomarkers more generally, may help to gain insight to which specific pathophysiological processes are driving disease progression and are targeted by a specific drug’s mode of action [31]. The combined losartan/irbesartan mechanism of action molecular model included a total of 48 protein coding genes retrieved from literature mining. Adding detailed omics profiling specifically on drug effect on kidney cells and tissue via in vitro or in vivo models would provide an improved representation of ARB molecular effect. While losartan and irbesartan are different molecules, we speculated that this would not affect the predictive ability of the serum metabolite classifier. Indeed, this appears to be the case as the classifier was able to predict albuminuria response in both losartan and irbesartan.

A key finding from the molecular process analysis is the relevance of nitric oxide (NO) in ARB response, as illustrated by the direct interference of ARB molecular mechanism of action and DKD molecular model by the enzyme NOS3, and further reflected by the inclusion of ADMA and citrulline in the serum metabolite classifier. ADMA, an endogeneous NO synthase inhibitor, is considered relevant in endothelial dysfunction contributing to extracellular matrix remodeling and being involved in inflammation [32]. Increased ADMA levels have been shown to contribute to increased risk of progressive DKD and predict fatal and nonfatal cardiovascular events in patients with type 1 diabetic nephropathy [33]. The positive beta values for ADMA in the regression models suggest that higher concentrations of the metabolite are associated with less albuminuria reduction. We therefore speculate that higher concentrations of ADMA lead to increased blockade of NOS3, resulting in a decrease in NO availability and diminished albuminuria response. Furthermore, citrulline, catalyzed by NOS3, has been shown to be inversely correlated to inflammatory parameters such as C-reactive protein [34]. Supplementation with citrulline has been shown to increase arginine/ADMA ratio, in turn decreasing blood pressure and improving vascular function [35]. Our study points to higher concentrations of citrulline associated with greater reductions in albuminuria. ADMA and citrulline assignments in the drug molecular model and DKD molecular model further suggest that albuminuria response to ARBs is reflected on the background of progressive disease as well as influenced by eNOS activity.

Some metabolites in the classifier could not be directly linked to the ARB mechanism of action molecular model, but were linked to processes driving progression of kidney disease. This indicates that in order to assess drug response, the interplay between disease progression molecular characteristics and specific drug molecular effects should be considered. This notion was further emphasized when testing a different subgroup of metabolites. The subset of seven metabolites being present in the DKD molecular model and being linked with oxidative stress response, inflammation and fibrosis (TGFB and downstream ECM remodeling) next to NOS3 activity significantly improved prediction of albuminuria response. Furthermore, interferences at the process units at the direct drug target together with the bradykinin system and on NFκB/PPARγ indicate involvement of inflammatory processes and lipid metabolism contributing to ARB response. These observations suggest that for assessing drug response, both disease progression status and specific drug molecular effects need to be taken into account. Metabolites of the renin-angiotensin-aldosterone system were not included in the classifier, suggesting that markers of RAAS activity do not predict the response to RAAS inhibition, which is in line with prior studies [36, 37].

Within the validation cohort, we were able to assess GFR change after the initial response period up to the end of follow-up. We observed an improvement in GFR over follow-up in patients who had a >30 % decrease in UAE from baseline, compared to a patients who did not see such a benefit in albuminuria response (Additional file 1: Table S2). This is in line with previous literature showing that greater reduction in albuminuria is associated with a lesser decline in eGFR during long-term follow-up [19]. Of interest, the metabolites were able to improve prediction of GFR changes, indicating that the serum metabolite classifier enables the identification of a group of patients who do not have a good albuminuria response to ARB therapy and have a fast deterioration of renal function over time. Further studies in large, diverse patient cohorts is necessary for validating these findings.

Limitations of this study include lack of comprehensiveness of drug mechanism of action characterization as the combined losartan/irbesartan mechanism of action molecular model was developed only from published literature. Undiscovered or unpublished mechanisms are therefore not represented in our model. Moreover, we were not able to determine the effect of co-medication in individual patients’ expression of metabolites. This would be important for further exploration in light of many (elderly) patients with diabetes mellitus with polypharmacy. In addition, the relative small sample sizes of the included trials may have decreased the precision of our effect estimates. Unfortunately, a type 2 diabetes validation cohort was not available. We therefore validated the metabolomics classifier in a type 1 diabetes cohort and demonstrated that the classifier was able to predict response to ARB therapy in this population as well. We acknowledge that the disease molecular mechanisms of type 1 diabetes and type 2 diabetes are very different from each other; however, we speculate that in terms of response to albuminuria lowering therapy, the response to treatment and predictors of response may be similar. We showed that the classifier was able to predict the albuminuria response to two different ARBs, but we were unable to assess within this study whether the metabolite classifier would be different in the same patient if that patient were taking a different class of RAAS blockade (such as ACEi). Whether the classifier predicts the response to other interventions in the RAAS has to be analyzed in future studies.

## Conclusions

In conclusion, we discovered and externally validated a classifier of 21 serum metabolites that significantly improve prediction of albuminuria response to ARBs in diabetes mellitus. Metabolites included in the classifier were assigned to stress/inflammation pathways and downstream consequences of fibrosis and extra cellular matrix remodeling. Specifically, NOS3 activity appears to be a specific factor relevant in ARB response. These results indicate that for assessing drug response, both disease progression status and specific drug molecular effects need to be taken into account. Moreover, the results of this metabolomics study support the growing evidence of using omics tools as a strategy to improve molecular characterization of drug effect and disease pathophysiology. The complementary use of omics platforms, integrated into molecular process models and from there determining biomarker panels, makes implementation of personalized medicine increasingly realistic in clinical practice.

## Additional file

10.1186/s12967-016-0960-3 Associations with baseline characteristics and change in UAE. **Table S2.** GFR change after response period in the validation cohort.

## Abbreviations

ADMA: asymmetric dimethylarginines
AGTR2: angiotensin II receptor, type 2
ARB: angiotensin receptor blocker
DKD: diabetic kidney disease
GFR: glomerular filtration rate
HbA1c: glycated hemoglobin
HMDB: human metabolome database
IDI: integrated discrimination improvement
IQR: interquartile range
KEGG: kyoto encyclopedia of genes and genomes
LASSO: least absolute shrinkage and selection operator
MSE: mean square error
NO: nitric oxide
NOS3: nitric oxide synthase 3
RAAS: renin-angiotensin-aldosterone system
ROC: receiver operating characteristics
SD: standard deviation
SBP: systolic blood pressure
UAE: urinary albumin excretion
