# Supplementary material for: Serum metabolites predict response to angiotensin II receptor blockers in patients with diabetes mellitus
Source: J Transl Med. 2016 Jul 5;14:203. doi: 10.1186/s12967-016-0960-3 (PMC4932762; doi:10.1186/s12967-016-0960-3)
Supplement: Supplementary file 1 — 10.1186/s12967-016-0960-3 Associations with baseline characteristics and change in UAE. Table S2. GFR change after response period in the validation cohort. [file 12967_2016_960_MOESM1_ESM.docx]

**Table S1.** Associations with baseline characteristics and change in UAE

|  | **Type 2 diabetes**  **Discovery cohort (n=49)** | | |  | **Type 1 diabetes**  **Validation cohort (n=50)** | | |
| --- | --- | --- | --- | --- | --- | --- | --- |
|  | β | S.E. | p-value |  | β | S.E. | p-value |
| Age at baseline | 0.58 | 0.53 | 0.281 |  | 0.8 | 0.9 | 0.386 |
| Male sex | 7.7 | 13.2 | 0.560 |  | -24.9 | 15.2 | 0.109 |
| Baseline SBP | -0.02 | 0.4 | 0.961 |  | 0.5 | 0.4 | 0.257 |
| Baseline DBP | -0.6 | 0.6 | 0.362 |  | 0.2 | 0.7 | 0.788 |
| Baseline HbA1c | 1.6 | 3.9 | 0.683 |  | 7.1 | 6.3 | 0.264 |
| Baseline Cholesterol | -0.8 | 5.4 | 0.877 |  | 4.4 | 7.6 | 0.567 |
| Baseline HDL | 19.4 | 15.8 | 0.225 |  | 14.0 | 14.3 | 0.332 |
| Baseline GFR | -0.2 | 0.3 | 0.554 |  | 0.02 | 0.3 | 0.961 |
| Baseline 24-hour logUAE | -7.9 | 5.7 | 0.177 |  | -12.6 | 9.1 | 0.174 |

**Table S2.** GFR change after response period in the validation cohort (n=50)

|  | ≤30% decrease in UAE | >30% decrease in UAE |
| --- | --- | --- |
| GFR change after response period (mL/min/1.73m^2^/year) | -5.2 (3.2) | -3.2 (3.7) |

Data are reported as mean ± standard deviation (SD).
